# Primary school mobile dental program in New South Wales, Australia: protocol for the evaluation of a state government oral health initiative

**DOI:** 10.1186/s12889-023-15241-6

**Published:** 2023-02-20

**Authors:** M Balasubramanian, A Ghanbarzadegan, W Sohn, A Killedar, P Sivaprakash, A Holden, S Norris, A Wilson, B Pogson, G Liston, L Chor, A Yaacoub, A Masoe, K Clarke, R Chen, A Milat, Carmen Huckel Schneider C

**Affiliations:** 1 Menzies Centre for Health Policy and Economics, Faculty of Medicine and Health, School of Public Health, The University of Sydney, Sydney, Australia; 2 Population Oral Health, Sydney Dental School, Faculty of Medicine and Health, The University of Sydney, Sydney, Australia; 3 Sydney Dental Hospital, Sydney Local Health District, NSW Health, St Leonards, NSW Australia; 4 Health Care Management, College of Business Government and Law, Flinders University, Adelaide, Australia; 5 Australian Research Centre for Population Oral Health, Adelaide Dental School, The University of Adelaide, Adelaide, Australia; 6 Centre for Oral Health Strategy, NSW Ministry of Health, St Leonards, NSW Australia; 7 Nepean Blue Mountains Local Health District, NSW Ministry of Health, Penrith, NSW Australia; 8 Centre for Epidemiology and Evidence, NSW Ministry of Health, St Leonards, NSW Australia; 9 School of Public Health, Faculty of Medicine and Health, The University of Sydney, Sydney, Australia

**Keywords:** Preventive care, Preventive Health Services, Mobile clinics, Oral health, Program evaluation, Quality of Health Care

## Abstract

**Background:**

Socioeconomically disadvantaged children are disproportionately affected by oral disease. Mobile dental services help underserved communities overcome barriers to accessing health care, including time, geography, and trust. The NSW Health Primary School Mobile Dental Program (PSMDP) is designed to provide diagnostic and preventive dental services to children at their schools. The PSMDP is mainly targeted toward high-risk children and priority populations. This study aims to evaluate the program’s performance across five local health districts (LHDs) where the program is being implemented.

**Methods:**

The evaluation will use routinely collected administrative data, along with other program-specific data sources, from the district public oral health services to conduct a statistical analysis that determines the reach and uptake of the program, its effectiveness, and the associated costs and cost-consequences. The PSMDP evaluation program utilises data from Electronic Dental Records (EDRs) and other data sources, including patient demographics, service mix, general health, oral health clinical data and risk factor information. The overall design includes cross-sectional and longitudinal components. The design combines comprehensive output monitoring across the five participating LHDs and investigates the associations between socio-demographic factors, service patterns and health outcomes. Time series analysis using difference-in-difference estimation will be conducted across the four years of the program, involving services, risk factors, and health outcomes. Comparison groups will be identified via propensity matching across the five participating LHDs. An economic analysis will estimate the costs and cost-consequences for children who participate in the program versus the comparison group.

**Discussion:**

The use of EDRs for oral health services evaluation research is a relatively new approach, and the evaluation works within the limitations and strengths of utilising administrative datasets. The study will also provide avenues to improve the quality of data collected and system-level improvements to better enable future services to be aligned with disease prevalence and population needs.

## Background

Oral health is fundamental to general health, well-being, and quality of life [1–3]. While evidence has shown significant improvements to the oral health of Australian children, these improvements have not been equitable across all population groups [4, 5]. Children from vulnerable and lower socioeconomic households and children living in rural and remote areas are disproportionately affected by oral disease and report access and financial challenges [5].

In Australia, tooth decay is the 7th leading cause of total disease burden among boys and the 4th among girls aged 5–14 years, accounting for 4.3% and 5.1% of the total burden of disease, respectively [4]. In 2012-14, approximately 42% of children in Australia had experienced tooth decay in their primary teeth and approximately 24% in their permanent teeth [6]. Prior evidence suggests a complex web of factors interact with a person’s oral health and their risk of developing tooth decay and other oral conditions such as gingival bleeding, gum disease, teething issues, and attrition/abrasion [2, 7]. Some of these factors include sugar consumption, oral health behaviours, exposure to fluoridated water, access to dental care, dental visiting patterns, insurance status, and affordability of dental care [7]. Early prevention and health promotion are vital in improving children’s oral health [8, 9].

### Health promotion in schools and mobile dental clinics

Historically, mobile clinics have played a vital role in the health care system, as they contribute to improving access and care provision for disadvantaged and vulnerable people [10, 11]. Prior literature has identified that mobile health clinics help underserved remote communities overcome barriers to accessing health care, including time, geography, and trust [10–15]. Mobile facilities providing general healthcare services in the USA have been shown to be associated with high rates of return on investment [15].

The philosophy of care provision in a school setting is strengthened by the Ottawa Charter of Health Promotion and is in line with the core principles of oral health promotion and community dentistry [16, 17]. Globally, in school-based programs offered has ranged from oral health promotion, education activities and preventive services to emergency oral health care. Schools offer a rich setting where care providers can effectively reach children during their early development phase and deliver oral health promotion, preventive education, and oral health interventions [17]. Prior research provides evidence of early school-based interventions’ longstanding effect on child oral health [18, 19]. Many types of providers have offered mobile dental care, ranging from philanthropic non-governmental not-for-profit organisations to organised private and public dental service providers.

### Primary school mobile dental program in New South Wales (NSW), Australia

Since the early 1970s, there have been several school dental programs funded by both Commonwealth and State governments [20, 21]. Services can range from oral health education visits such as tooth brushing techniques, developing good oral health habits and dental interventions (mostly diagnostic and preventive). Similar programs also exist in neighbouring states such as Victoria [22] and South Australia [23]. Outreach based mobile school dental programs have proven to be effective in improving health outcomes and provide greater value for the dental dollar [24–26].

In July 2019, the Government of NSW, a state in Australia, initiated the NSW Health Primary School Mobile Dental Program (PSMDP), which is intended to allow access to oral health care for up to 136,000 primary school children annually, with a commitment of $70 million across the four years of the program commencing 2019 [27, 28]. Five local health districts (LHDs), that are responsible for delivery of public health services, involved in this program: Central Coast, Mid North Coast, Nepean Blue Mountains, South Western Sydney, and Western Sydney LHDs [27]. The first selection of schools in the program was based on the socio-educational background of students, with priority given to schools with low Index of Community Socio-Educational Advantage (ICSEA) scores. The program was planned to be delivered by the public oral health teams.

The PSMDP is designed primarily to provide diagnostic and preventive services to primary school children via a mobile dental clinic [27, 29], however, implementation models differed slightly between the LHDs as they have been adapted to local needs and oral health resources. Schools are approached by individual LHDs for participation in the PSMDP. In schools that agree to participate, parents and carers receive a consent form and risk factor questionnaire prior to the actual visit of the oral health team to the school [29]. Children who have consented to participate in the program can receive the following types of care (dependant on the child’s risk status): a comprehensive examination, dental x-rays, dental scale and clean, fluoride varnish application, and pit and fissure sealant application [27]. In some LHDs the PSMDP also provides treatments including urgent and restorative services. Review appointments may be scheduled if needed. If further treatment including urgent care is required children may be referred to a fixed local public dental service.

The PSMDP intends to provide high-quality, efficient, effective, and safe oral health preventive care and treatment for primary school children in a way that is acceptable to children, parents, and schools. This program aims to increase access to comprehensive oral health services for primary school children in target regions, particularly high-risk children and priority populations. It is also anticipated that the program will help coordinate more complex treatment, including specialist dental care, with local public and private dental providers.

## Study aims

The goal of this analysis is to evaluate the Primary School Mobile Dental Program in New South Wales, Australia. The study will include all five LHDs, where the program is being implemented from 2019 to 2023. The analysis will include assessment of the program’s reach, uptake, outcomes, and cost, and will involve both cross-sectional and longitudinal analysis.

### Study aim 1: reach and uptake of the program

- To determine the number, scope and extent of services being offered to target schools and children across the five participating LHDs.
- To examine the extent to which the program is utilised by schools, parents, and children across the five participating LHDs.
- To explore the nature and type of services being delivered at the mobile dental facilities and services being followed up at fixed dental clinics across the five participating LHDs.
- To analyse trends over time in the provision and uptake of services across the program duration.

### Study aim 2: health outcomes and risk factors

- To assess oral and general health outcomes, oral health behaviours, and dental visiting patterns of children enrolled in the program across the five participating LHDs.
- To investigate associations between children’s background characteristics, services received, oral and general health outcomes, oral health behaviours and dental visiting patterns.
- To examine changes in health outcomes, oral health behaviours and health service utilisation of enrolled children.

### Study aim 3: effectiveness, costs, and cost-consequences

- To estimate the effectiveness of the program by comparing oral health outcomes for children who participate in the program versus matched, non-participating children across the five LHDs.
- To estimate the financial costs of establishing and running the program by year and by LHD.
- To estimate the costs of non-program oral health services for children participating versus non-participating (i.e. the consequences of receiving or not receiving initial care via the program).

## Methods

The study’s overall design combines comprehensive output monitoring in five participating LHDs, along with an analysis of associations between socio-demographic factors, risk factors, participation in the program, treatments received, and health outcomes. Time series analysis using difference-in-difference estimation will be conducted across the first four years of the program involving services, risk factors and health outcomes [30]. Comparison groups will be identified via propensity matching across the participating LHDs [31, 32]. An economic analysis will be conducted to estimate the costs and cost-consequences of the program [33, 34].

### Target populations

**Fig. 1:**
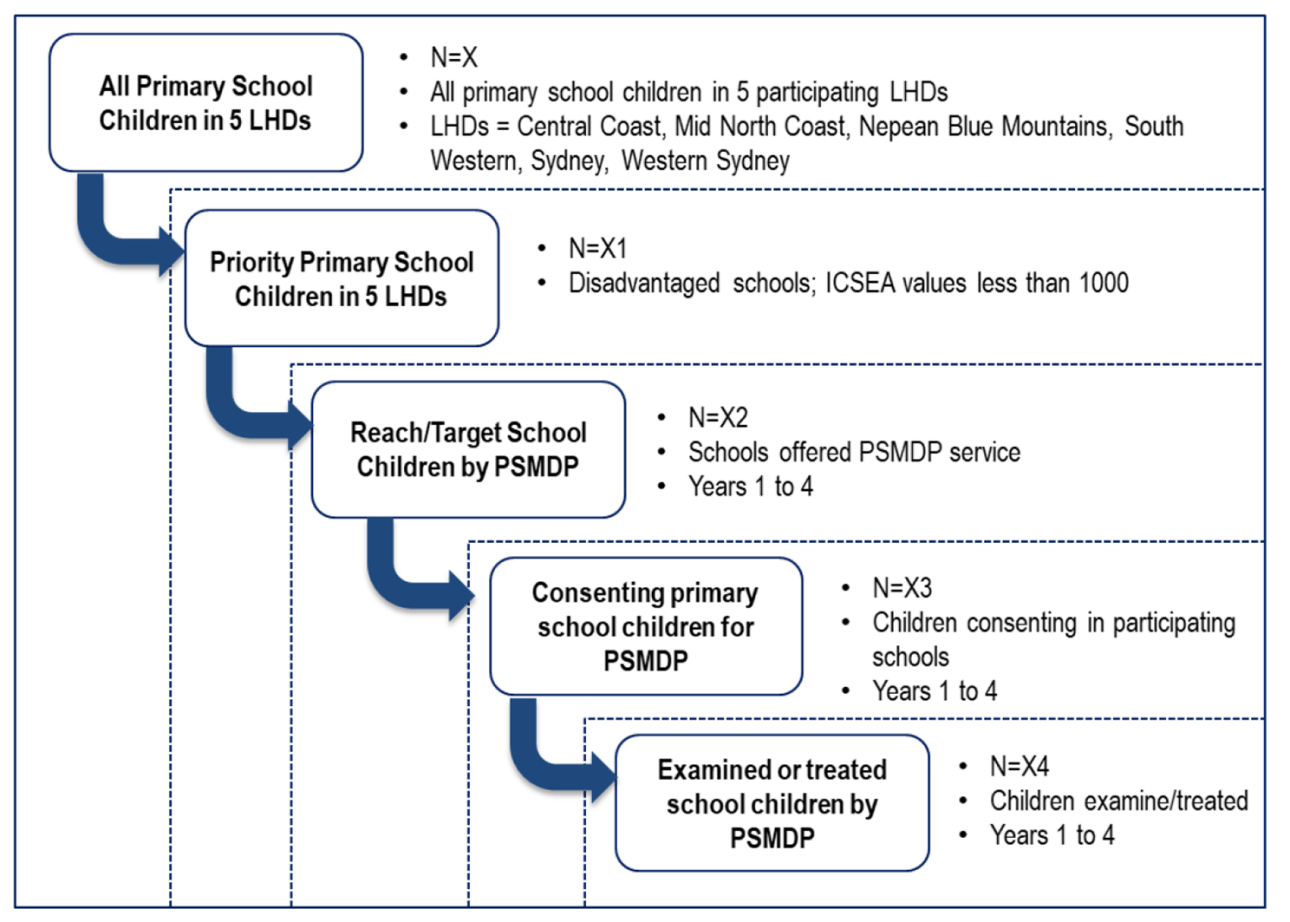
Conceptual Target Populations

Figure 1 illustrates the conceptual target populations of the PSMDP at five levels. At the upper level are all primary school children at public schools in the five LHDs (N = X). This is the whole target population of the PSMDP. The second level are priority primary school children (N = X1), i.e., children in schools with an Index of Community Social-Educational Advantage (ICSEA) value less than 1000. Children in schools targeted by the program and who receive service visits are classified as being offered^1^ services (N = X2). It is expected that the number of schools being offered the program will gradually increase throughout the program. Not all children in the schools being offered the program have received parental consent to take part in the program. The third level includes children in the schools with parental consent (N = X3). At the inner level are those children with parental consent who attend the PSMDP and receive a package of services (N = X4). The package of services usually during the visits to the school. Follow-up services may also be received at a fixed public dental clinic according to the model of care available within each LHD. It is not mandatory under the program for children to be followed every year. Whilst some children will re-present/re-engage with the program, not all children are required to be followed up during the program.

### Data sources

Data from three main sources will be used in the project: Electronic Dental Records (EDRs); Community Oral Health Program Portal data (COHPP); and Risk Factor Data. EDRs are widely used in NSW public dental services, and since April 2018, LHDs have upgraded their systems to new Electronic Dental Record System ‘Titanium’ to record a wide range of patient demographic, patient flow and service delivery data [1, 35, 36]. LHDs are required to enter necessary patient, provider, and service information at the point of care including at the mobile dental facility, which is then centrally available in the Titanium EDRs database. The Community Oral Health Program Portal (COHPP) collects scheduling information on school visits by the mobile dental facility. This system manages schools and consent information. The scheduling system collects information on schools and children enrolled, dates, and visits to each school. Data is sourced from a manual scheduling system which is available as spreadsheet information, and/or an integrated electronic system for managing visits by the mobile school facility. The risk factor data is available as part of the consent and information pack forms completed by each participating parent/child prior to enrolment in the program. All children participating in the program, irrespective of the participating year(s), record risk factor and consent data. This information is either available separately as .*csv* files or integrated into the EDRs system. Additional financial data is collected and reported directly from the MoH or individual LHDs.

### Data items

Some data items are specific to each source and a few are available in more than one source. Table 1 provides a summary of these data items and data sources. Data from all three sources will be combined, where possible, for analysis. Data will be sourced for all the first four years of the program.

**Table 1:** Data categories, data items and data sources

No.	Category	Data item	Data source
1	Demographic data	Age; Gender; Country of Birth; Indigenous status; Language spoken at home; Name of school enrolled; school class	Titanium EDRs; Risk factor dataset
2	Socioeconomic data	Postcode of residency; health card status; Medicare status	Titanium EDRs; Risk factor dataset
3	Schools’ data	School enrolled; Name of school visited; date of visit; number of schools visited; postcode of school; consents received	COHPP; Risk factor dataset; Titanium EDRs
4	Clinical dental data	DMFT/dmft, Number of teeth present	Titanium EDRs
5	General health data	Weight, height; medications; allergies; bleeding problems; heart condition; blood pressure; respiratory condition; mental health; disability	Titanium EDRs; Risk factor dataset
6	Oral health utilisation and treatment data	Mobile dental facility visits (name of facility, LHD); Date of visit to the mobile dental facility; Services offered at the mobile dental facility; service code and categories; provider information at the mobile dental facility; referral or follow-up at the fixed dental facility; provider information at follow up facility; prior visits to public dental service	Titanium EDRs
7	Dental history	Prior dental problems; prior visit to dental professional; type of dental visit;	Risk factor dataset; Titanium EDRs;
8	Oral health behaviours	Sugar consumption; water consumption; brushing habits; toothpaste use	Risk factor dataset
9	Quality of life	Self-rated oral health	Risk factor dataset
10	Other clinically relevant data	Clinical conditions (periodontal status, plaque, saliva, etc.)	Titanium EDRs

#### Electronic Dental Records

The following secondary data will be sourced from Titanium EDRs [36]: patient clinical (treatment, oral health and medical history) and background information (such as demographic and socioeconomic data). Key oral health clinical measures such as decayed, missing and filled teeth (DMFT/dmft), number of teeth present and general health measures such as height, weight, and medical conditions. Service mix and provider information such as dental visits at a mobile dental facility, date and frequency of visit, services offered at the mobile dental facility, provider data, referral or follow-up at a fixed dental facility, services offered at a fixed dental facility and prior visits to any public dental facility will be obtained from the Titanium database.

#### Community Oral Health Program Portal data

Data on schools contacted, schools which accepted to participate in the program, date of visits, total school enrolment, consents rate, and children receiving care in the program will be sourced from the COHPP.

#### Risk factor dataset

The risk factor survey provides data on the socio-demographic profile of children, oral health behaviours (tooth brushing habits, sugar and water consumption, toothpaste use), oral health outcomes (self-rated oral health), dental visiting patterns and medical information. This information will be sourced from the consent forms and risk factor questionnaire data available from the COHS team.

### Comparison groups

Based on our conceptual target populations (Table 1), comparison groups will be drawn from non-participating children among all eligible children across the 5 LHDs. Using data from the Titanium EDRs, comparison groups will be identified via propensity matching to ensure comparability between participating and non-participating children. Clinical data from Titanium EDRs will enable assessment of differences between participating and non-participating children across the five LHDs, and throughout the program.

### Data management

De-identified data from the three secondary data sources will be made available to the study team, following NSW Health data transfer protocols. Combined datasets will be transferred via the secure data transfer system of NSW Health and downloaded using University of Sydney computers based at the Menzies Centre for Health Policy and Economics. Data transfer will occur once every three months during the program. Data will be taken to a statistical package (R/RStudio) available in existing computer facilities at the University of Sydney. Data consistency checks will be conducted; range checks and cross-tabulations will be performed initially to ensure data is correct. Data cleaning will be undertaken to ensure the usability of the data. Variables will be conventionally named along with definitions, and a data dictionary will be prepared prior to analysis. Microsoft PowerBI (licensed by the University of Sydney) will be used for dashboards and visualisation, along with the statistical packages.

### Data analysis plan

#### Study aim 1: reach and uptake of the program

Analysis for study aim 1 will be guided by an understanding of the population subsets eligible, reached, consented, and examined/treated in the program (Fig. 1). Descriptive data analysis will be conducted annually for monitoring and surveillance requirements on the reach and uptake of services. Patient (socio demographic data, previous history), provider (service facility, practitioner type), schools (participation, visits), and service (service provision) characteristics will be examined. Summary data will be reported on patient and provider characteristics, schools visited, children offered service, consent rates, examinations, follow-ups, and service mix. Reporting will be through number (n), proportions (%) and trends.

Dental treatment codes will be aggregated into available service types such as restorative, diagnostic, preventive, extraction, endodontic, and restorative for the purposes of reporting. Services provided at the mobile dental facility, and characteristics will be examined across available provider and service types. Service mix data will be reported in rates across patient and provider characteristics, service category and LHD. Time trends will be investigated across the duration of the program. Trends will be explored across patient, provider, and service characteristics to demonstrate changes in the reach and uptake of the program across the five participating LHDs.

#### Study aim 2: health outcomes and risk factors

**Fig. 2:**
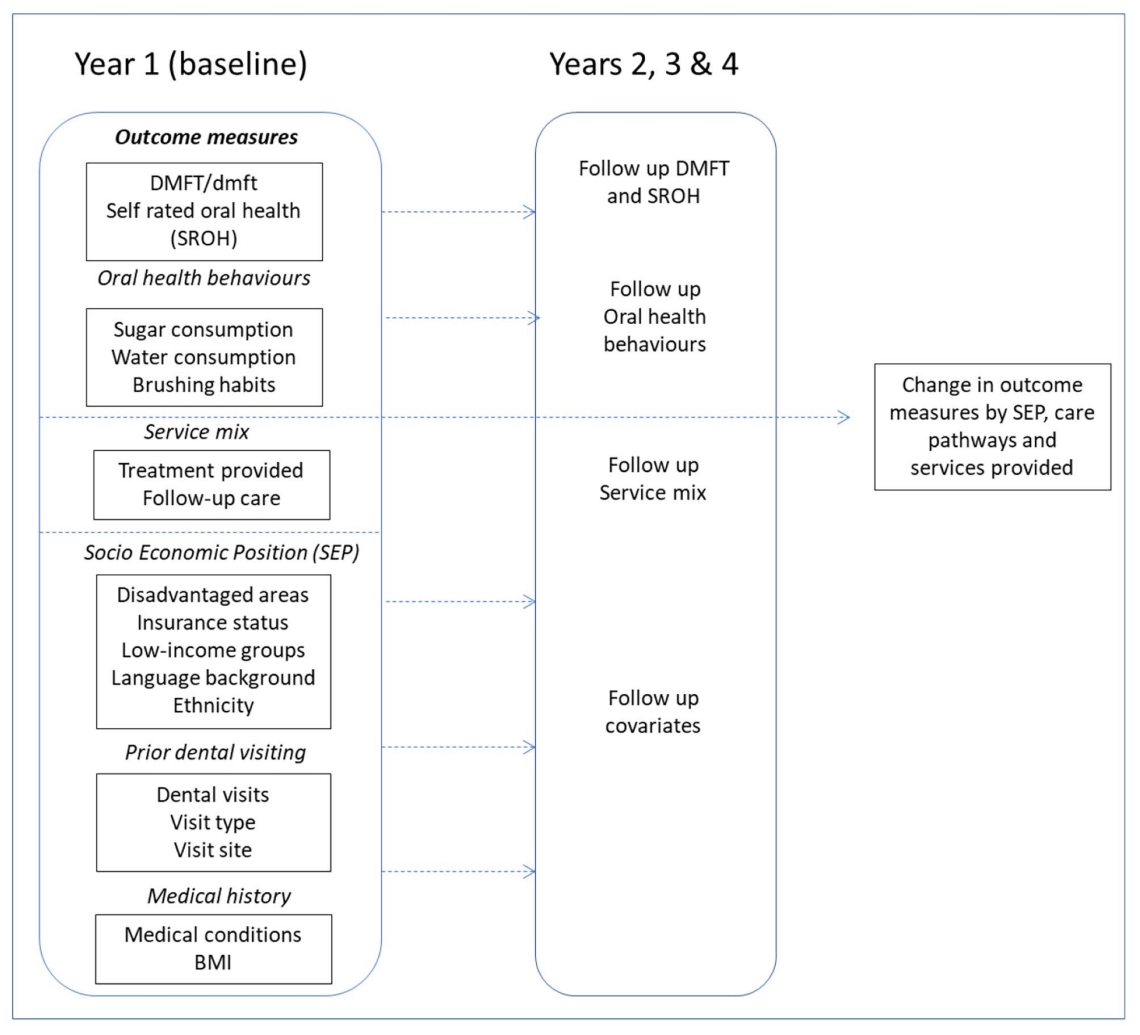
Overview of the analysis plan

The analysis for the study will describe and test differences in outcome variables in relation to specific explanatory variables. Inferential analysis will examine bivariate associations and potential joint effects before multivariate analysis, which will control for a range of covariates and potential confounders such as age, gender, and socioeconomic status. The broad goal of this analysis is to explore if participation in the PSMDP contributes to a difference in health outcomes (including self-reported oral health, and other oral health outcome variables such as DMFT/dmft). The overarching study design seeks to compare the oral health outcomes over time for the PSMDP participants and non-participants, who are in similar geographical regions, and schools; and also determine the impact of various services provided on their oral health outcomes. By including other multiple explanatory data measures, as outlined in Fig. 2, we will seek to account for possible confounders.

Outcome measures: The main oral health clinical outcome measure is decayed missing filled teeth (DMFT/dmft). DMFT/dmft data will be extracted from Titanium EDRs, for the duration of the program. Overall DMFT scores will be used as an aggregate measure. Changes in DMFT will be analysed individually for the change in decayed, missing and filled status of the tooth surface. Self-Rated Oral Health (SROH) will be extracted from the risk factor dataset. SROH is captured in a five-point Likert scale ranging from poor to excellent. The variable will be dichotomised, when appropriate, prior to the bivariate and multivariate analysis. All oral health behaviours will be dichotomise as being healthy or unhealthy habits before being stratified in the bivariate and multivariate analysis. Oral health behaviours include sugar and water consumption and tooth brushing habits. This data will be extracted from the risk factor dataset.

##### Explanatory variables

Services provided in the PSMDP will be extracted as service codes from the Titanium dataset. Service codes will be aggregated into various categories. Analyses will include a particular service (provision of topical fluoride application) and/or by service categories (e.g., restorative services, preventive services). Service provision by provider categories, i.e., if they have received service at a mobile dental facility or fixed dental facility, type of provider or model of care will be analysed. For the comparison groups, services provided in a public dental facility will be sourced from Titanium EDRs.

##### Covariates

Demographics (including age, gender, aboriginality and language background other than English), socioeconomic and schools background data will be analysed as covariates. Socioeconomic position will include disadvantaged areas, health care status, income categories, language background, and ethnicity. Schools will be categorised according to NSW Department of Education stratifications [37].We will use the Australian Standard Geographic Classification (ASGC) Remoteness Index and Socio-Economic Index for Areas - Disadvantaged (SEIFA) to provide categories on remoteness and disadvantage based on the residency area [38, 39]. Country of birth will be categorised according to World Health Organization (WHO) and World Bank regions.

#### For study aim 3: costs and cost-consequences

##### Time-series

Difference-in-Differences analysis.

A time-series analysis of data on services provided, referrals, oral and general health indicators, oral health outcomes, oral health behaviours and health services utilisation patterns will be undertaken across the duration of the program. A difference-in-differences (DID) analysis will use longitudinal data from treatment and comparison groups. We will estimate an appropriate counterfactual to determine the causal effect. The DID analysis method is typically used in situations where changes in outcome over time between the population that is enrolled in a program, and that is not enrolled in a program [30]. Propensity score matching [32] will be used to determine the comparison groups for the study.

##### Economic analysis

The costs and consequences of the program will be determined from the perspective of the health funder (NSW Health) and from a societal perspective. Costs will be expressed in Australian dollars (deflated to 2019 values, the first year of the program), and will be reported by LHD and by year of the program. An annual discount rate of 7% will be applied in the base case (with sensitivity analyses at 3 and 10%; as specified by the NSW Government) [40].

From the health funder perspective, the costs associated with delivering the program over the first four years will include: capital outlays and recurrent expenditure (e.g., maintenance of capital, staffing, travel and transport costs, consumables, insurance), and any other services or capital provided in-kind from within the NSW Health portfolio. From the societal perspective, the costs associated with delivering the program will additionally include resource use from outside the NSW Health portfolio, namely teachers’ and school administrators’ time to organise the program in each school, and parent/carer time off work to attend dental visits for participating children.

The cost consequences per child in the treatment and comparison groups will be estimated by applying appropriate public sector unit costs from the Child Dental Benefits Schedule (CDBS) to the profile of oral health service use observed in each group. Simplifying assumptions will be made regarding patient eligibility for the CDBS and the conditions under which the patient benefit cap is applied.

Sensitivity analyses will be conducted around key parameters, including variance in the observed data described above, and any assumptions made when estimating costs (e.g., salary scales used to value teachers’ time; or methods used to estimate the lost productivity of parents/carers when attending dental appointments for their child).

## Discussion

The study seeks to use routinely collected clinical and administrative data from the LHD public oral health services, including the program, to conduct analysis that determines the reach and uptake of the program, its effectiveness, outcomes and the associated costs and cost-consequences. The design of the study is pragmatic, considering factors such as availability of data, acceptability of study to stakeholders, and challenges associated with real world evaluation.

### Limitations

Using Electronic Dental Records (EDRs) for oral health services evaluation research is a relatively new approach, and some challenges and limitations arise in using administrative datasets. While Titanium EDRs for public dental services provides a comprehensive suite of applications for capturing patient demographics, service mix, appointment history, provider details and clinical data, the adoption, implementation, and usage of Titanium EDRs across LHDs has not necessarily been a uniform process across all five LHDs. Further, using any electronic health record system requires appropriate training to leverage its potential, as well as supportive information systems, management practices and health services expertise to facilitate the translation of data into meaningful evidence to guide clinical, management and policy decisions [41, 42]. The study will be constrained to the different levels of information available from the EDRs, and what is comparable across the five LHDs.

Titanium EDR has a DMFT/dmft function which has the capacity to automatically translate the clinical chart coding to DMFT/dmft scores. However, this function is not turned on by default, and LHDs have opted to not turn on the DMFT/dmft function within Titanium due to various reasons (such as time constraints and technology limitations). Consequently, the option for this study is to determine DMFT/dmft score via a post hoc measurement algorithm, specific for the PSMDP program using clinical chart coding and service mix codes. Within NSW Health, the DMFT/dmft metric is taken from clinical records and therefore has some technical and clinical limitations within the data that form the EDRs. Thus, the post hoc estimation of DMFT/dmft presents some limitations in the number of cases with full DMFT/dmft status being recorded, and thus the DMFT/dmft is a likely subset of the overall analysis.

The program serves children across participating schools in five LHDs in NSW. A limitation is while schools can be potentially visited consecutively across the first four years of the program, this would be dependent on operational issues at the schools and across each LHD. Therefore, follow up of children is not necessarily uniform across the years of the program, providing some limitations for the longitudinal analysis.

This protocol paper presents the quantitative aspect of the evaluation, incorporating data from the three main source (EDRs, risk factor data and scheduling data). A few additional components including qualitative interviews and understanding process of implementation and operationalising of the program are separate qualitative studies outside this protocol paper. A considerable limitation on the program and its evaluation is that the COVID-19 pandemic has an impact on the operational scope and economic cost of the program. The COVID-19 pandemic has resulted in some disruption to the program either directly during school closures or indirectly by limiting access, uptake from schools and parents or limitation related to the workforce availability. Assuredly, the pragmatic design of this real-world evaluation brings the flexibility to address these issues during the evaluation.

## Conclusion

The paper presents the protocol for the quantitative aspects of the performance evaluation of the four-year state government health transformation plan, the PSMDP. By quantifying the reach and effectiveness of the program using a range of measures it is anticipated that the findings of the evaluation will be considered to inform program improvements and scale-up across NSW, with the ultimate goal of improving access to effective oral health services for children across the state. It is anticipated that the study will also identify opportunities to improve the quality of data collected, system-level improvements, as well as refining future services so they are better aligned with disease prevalence and population needs.

## Data Availability

NA.

## Footnotes

1 Offerred indicates provided the oppurtunity to take part in the program, considered as program reach.

## List of abbreviations

ASGC: Australian Standard Geographic Classification
CDBS: Child Dental Benefits Schedule
COHPP: Community Oral Health Program Portal
COHS: Centre for Oral Health Strategy
dmft: Decayed Missing Filled (deciduous) Teeth
DMFT: Decayed Missing Filled (permanent) Teeth
PSMDP: Primary School Mobile Dental Program
EHRs: Electronic Health Records
EDRs: Electronic Dental Records
ICSEA: Index of Community Socio-Educational Advantage
ISOH: Information System for Oral Health
NSW: New South Wales
NHMRC: National Health and Medical Research Council
LHD: Local Health District
SROH: Self-Rated Oral Health
SEIFA: Socio-Economic Index for Areas
